# Identification and Functional Analysis of Delta-9 Desaturase, a Key Enzyme in PUFA Synthesis, Isolated from the Oleaginous Diatom *Fistulifera*

**DOI:** 10.1371/journal.pone.0073507

**Published:** 2013-09-05

**Authors:** Masaki Muto, Chihiro Kubota, Masayoshi Tanaka, Akira Satoh, Mitsufumi Matsumoto, Tomoko Yoshino, Tsuyoshi Tanaka

**Affiliations:** 1 Division of Biotechnology and Life Science, Institute of Engineering, Tokyo University of Agriculture and Technology, Koganei, Tokyo, Japan; 2 JST, CREST, Sanbancho 5, Chiyoda-ku, Tokyo, Japan; 3 BT Development Group, Research and Development Section, Technology Center, Yamaha Motor Co. Ltd., Fukuroi, Shizuoka, Japan; 4 Biotechnology Laboratory, Electric Power Development Co. Ltd., Yanagisaki-machi, Wakamatsu-ku, Kitakyusyu, Japan; University of South Florida College of Medicine, United States of America

## Abstract

Oleaginous microalgae are one of the promising resource of nonedible biodiesel fuel (BDF) feed stock alternatives. Now a challenge task is the decrease of the long-chain polyunsaturated fatty acids (PUFAs) content affecting on the BDF oxidative stability by using gene manipulation techniques. However, only the limited knowledge has been available concerning the fatty acid and PUFA synthesis pathways in microalgae. Especially, the function of Δ9 desaturase, which is a key enzyme in PUFA synthesis pathway, has not been determined in diatom. In this study, 4 *Δ^9^ desaturase* genes (*fD9desA*, *fD9desB*, *fD9desC* and *fD9desD*) from the oleaginous diatom *Fistulifera* were newly isolated and functionally characterized. The putative Δ^9^ acyl-CoA desaturases in the endoplasmic reticulum (ER) showed 3 histidine clusters that are well-conserved motifs in the typical Δ^9^ desaturase. Furthermore, the function of these Δ^9^ desaturases was confirmed in the *Saccharomyces cerevisiae ole1* gene deletion mutant (*Δole1*). All the putative Δ^9^ acyl-CoA desaturases showed Δ^9^ desaturation activity for C16∶0 fatty acids; fD9desA and fD9desB also showed desaturation activity for C18∶0 fatty acids. This study represents the first functional analysis of Δ^9^ desaturases from oleaginous microalgae and from diatoms as the first enzyme to introduce a double bond in saturated fatty acids during PUFA synthesis. The findings will provide beneficial insights into applying metabolic engineering processes to suppressing PUFA synthesis in this oleaginous microalgal strain.

## Introduction

Biodiesel fuel (BDF) has attracted considerable attention over the past decade as a renewable and biodegradable fuel alternative to fossil fuels. Commercially available BDFs are produced from a variety of terrestrial plants, including soybean, rapeseed, sunflower, castor seed, jatropha and palm oil. Terrestrial plants potentially have a negative impact on food supply. Furthermore, they have lower oil yield per area than oleaginous microalgae [1], [2], [3]. Based on the reasons, recently, oleaginous microalgae have been intensively studied as non-food biomass and high-triacylglyceride (TAG) producer for efficient BDF production [1], [4].

BDF is a series of fatty acid methyl esters (FAMEs) generated by transesterification of TAG from feedstocks [5]. The physical and chemical properties of FAMEs are determined by its acyl composition, with respect to both carbon chain length and the number of double bonds. As the degree of unsaturation of fatty acids in FAMEs particularly affects the oxidative stability of BDF [6], the unsaturated fatty acid content in BDF is a primary limitation to its commercial use [7]. BDFs from soybean, sunflower and grape seed contain high levels of polyunsaturated fatty acids (PUFAs), resulting in poor oxidative stability [8], [9]. On the other hand, BDFs from rapeseed, olive, corn, almond and high oleic sunflower oils show superior BDF properties because of their high content of monounsaturated [8]. Microalgal TAG mainly consists of short and saturated fatty acids, however, non-negligible quantities of long-chain PUFAs, such as methyl linolenate (C18∶3), eicosapentaenoic acid (EPA; C20∶5) or docosahexaenoic acid (DHA; C22∶6) are also involved [10], [11].

Toward addressing the above issue, breeding efforts have been done in terrestrial plants. The PUFA contents of TAG have been successfully reduced by the suppression of *desaturase* gene expression using RNA interference (RNAi) system in soybean, cotton seed and brassica seed [12], [13], [14]. By contrast, in microalgae, genetic modifications of FAME profiles have been hampered by the limited knowledge available concerning the fatty acid synthesis pathway (including PUFA synthesis) and/or by difficulties in the genetic engineering approach [15], [16]. Among eukaryotic microalgal groups, diatoms are well-established in terms of genomic and transgenic capabilities. Furthermore, the enzymes involved in fatty acid synthesis have been primarily identified in a model diatom, *Phaeodactylum tricornutum* [17], [18], [19]. In *P. tricornutum*, ω3, Δ5, Δ6 and Δ12 desaturases were responsible for PUFA synthesis [18], [19]. The end-product of the pathway is EPA. However, among various desaturases, the function of Δ9 desaturase from diatom has not been determined, although the enzyme plays a key role in PUFA synthesis as the first enzyme to introduce a double bond into saturated fatty acids [19].

A marine oleaginous diatom, *Fistulifera* sp. used in this study, has been recognized as a potential candidate for BDF production [20] because of its exceedingly high levels of intracellular TAGs (60% w/w) and its rapid growth. High-cell-density cultivation and outdoor mass cultivation of *Fistulifera* sp. have been demonstrated in flat-type photobioreactors [21], and column-type and raceway-type bioreactors [22]. In this strain, the major fatty acids are palmitate (C16∶0; 30–40% of total fatty acids), palmitoleate (C16∶1; 40–50%) and eicosapentaenoic acid (EPA, C20∶5; 4–20%) as a PUFA. Recently, genetic transformation for this strain was performed [23]. Metabolic engineering with the gene manipulation technique is a promising approach to decrease the PUFA content in TAG. One of the targets for genetic transformation was Δ9 desaturase because they may play a key role in fatty acid (and subsequent TAG) synthesis [12], [13], [14].

In this study, we report the screening of *Δ^9^ desaturase* genes in the oleaginous diatom *Fistulifera* and their functional characterization by expression in the yeast *Δole1* mutant. Through the comparison of the isolated Δ^9^ desaturases with those from other diatoms, unique features of *Δ^9^ desaturase* genes in *Fistulifera* sp. were determined. To our knowledge, this is the first study to confirm the function of Δ^9^ desaturases in diatoms and also in oleaginous microalgae.

## Materials and Methods

### Strains and Growth Conditions

The marine pennate diatom *Fistulifera* sp. was grown in half-strength Guillard’s “f” solution (f/2) [24] dissolved in artificial seawater (Tomita Pharmaceutical Co. Ltd., Naruto, Japan). Cultures were grown at 25°C under continuous and cool-white fluorescent lights at 140 µmol**·**m^−2^ **·**s^−1^ with aeration. Genes were cloned in *Escherichia coli* TOP10 (Invitrogen, Carlsbad, CA, USA) or *E. coli* DH5α (BioDynamics Laboratory Inc., Tokyo, Japan) cultured in Luria broth (Merck, Darmstadt, Germany) containing 50 µg/mL kanamycin or ampicillin at 37°C.

Putative *Δ^9^ desaturase* genes were expressed in *Saccharomyces cerevisiae* INVSc-1 (*MATa/MATα, his3Δ1/his3Δ1, leu2/leu2, trip1-289/trip1-289,* and *ura3-52/ura3-52*) (Invitrogen) or the yeast *Δole1* mutant (*MATα, his3Δ1, leu2Δ0, ura3Δ0,* and *ole1Δ::kanMX4*) [25]. The yeast *Δole1* mutant (*MATα, his3Δ1, leu2Δ0, ura3Δ0,* and *ole1Δ::kanMX4*) was generated via the sporulation of the *S. cerevisiae* YGL055W/BY4743 heterozygous strain (*MATa/MATα, his3Δ1/his3Δ1, leu2Δ0/leu2Δ0, lys2Δ0/+, met15Δ0/+, ura3Δ0/ura3Δ0,* and *ole1Δ::kanMX4*) (ATCC number: 4024422).

### Isolation of *Δ^9^ desaturase* Genes from *Fistulifera* sp

To obtain the putative *Δ^9^ desaturase* genes of *Fistulifera* sp., a homology search using BlastX was performed with reference to the 19,859 genes from the draft genome sequence of *Fistulifera* sp. [26]. The full-length cDNAs of putative *Δ^9^ desaturase* genes were obtained by 5′- and 3′-RACE using a Smarter RACE cDNA amplification kit (Clontech, Palo Alto, CA, USA). Partial sequences of these genes predicted by the AUGUSTUS program were used for designing gene-specific primers to amplify the 5′ and 3′ ends of the target genes (Table S1). The PCR products were cloned into the pCR-Blunt II-TOPO vector (Invitrogen). The full-length cDNA sequences were assembled based on the 5′- and 3′-RACE fragments.

### Sequence Analysis

Amino acid sequence alignments of Δ^9^ desaturases from different organisms were generated using the ClustalW program (http://www.genome.jp/tools/clustalw/). The phylogenetic tree was constructed via the neighbor-joining method and evaluated with 1,000 rounds of bootstrapping using MEGA4. Δ^9^ desaturase amino acid sequences were retrieved from the databases of the whole genome of *P. tricornutum* [17] and *Thalassiosira pseudonana* [27] by a BlastX search using the *Δ^9^ desaturase* genes of *Fistulifera* sp. as the query sequence. Cloned sequences and other putative diatom sequences were also investigated to determine whether the protein has N-terminal signal peptides; SignalP 4.0 (http://www.cbs.dtu.dk/services/SignalP/) [28], TargetP 1.1 (http://www.cbs.dtu.dk/services/TargetP/) [29], and HECTAR (http://www.sb-roscoff.fr/hectar/) [30] were used for this analysis. TMHMM 2.0 (http://www.cbs.dtu.dk/services/TMHMM) [31] and TMHTOP 2.0 (http://www.enzim.hu/hmmtop/index.php) [32] were used for the prediction of transmembrane domains.

### Functional Characterization of *Δ^9^* Desaturases in the Yeast *Δole1* Mutant

For functional characterization, 4 *Δ^9^ desaturase* genes (*fD9desA*, *fD9desB*, *fD9desC*, and *fD9desD*) with the Kozak sequence [33] in front of the start codon were cloned into the yeast expression vector pYES2.1/V5-His-TOPO (Invitrogen) (Table S1). The yeast *Δole1* mutant was transformed with plasmid DNA with a polyethylene glycol/lithium acetate protocol [34]. The yeast cells harboring the control pYES2.1/V5-His/*lacZ* were used as a negative control. All transformants were selected by uracil prototrophy on a selective dropout media (SD) plate lacking uracil. For functional expression, SD medium containing 2% (w/v) galactose, 1% Tergitol Type NP-40 (Invitrogen), and 500 µM C16∶1 or C18∶1 fatty acids was inoculated with the pYES2.1FsDES9 transformants and grown at 20°C for 96 h in a water bath shaker. Cell pellets were sequentially washed with 1% Tergitol Type NP-40 and 0.5% Tergitol Type NP-40, freeze-dried, and subject to fatty acid analysis.

### Complementation Assay in the Yeast *Δole1* Mutant

Each transformant harboring the plasmid for the expression of Δ^9^ desaturases was suspended in distilled water and adjusted to an OD_600_ of 1 and 0.1. The resulting 2.5 µL yeast solution was spotted on an SD agar plate lacking uracil but containing 2% galactose and (a) no fatty acids; (b) 500 µM C16∶1 fatty acid and 1% Tergitol Type NP-40; or (c) 500 µM of the C18∶1 fatty acid and 1% Tergitol Type NP-40. Cell growth was evaluated after 72 h at 30°C to examine the unsaturated fatty acid requirement for the growth of yeast *Δole1* mutant transformants.

### Fatty Acid Analysis Using GC/MS

The freeze-dried yeast cells were directly transmethylated with 1.25 M hydrochloric acid in methanol (1 h at 100°C) to prepare the FAMEs. The FAMEs were extracted in *n-*hexane and analyzed by GC/MS (QP2010 Plus; Shimadzu, Kyoto, Japan) with FAMEWAX (RESTEK, Bellefonte, PA, USA) in the electron impact mode. FAMEs were identified using the F.A.M.E. Mix, C4–C24 Unsaturates (Sigma-Aldrich, Dorset, UK). Each sample was analyzed in 3 independent experiments.

## Results

### Sequence Analysis of Putative *Δ^9^ Desaturase* Genes from *Fistulifera* sp

**Figure 1:**
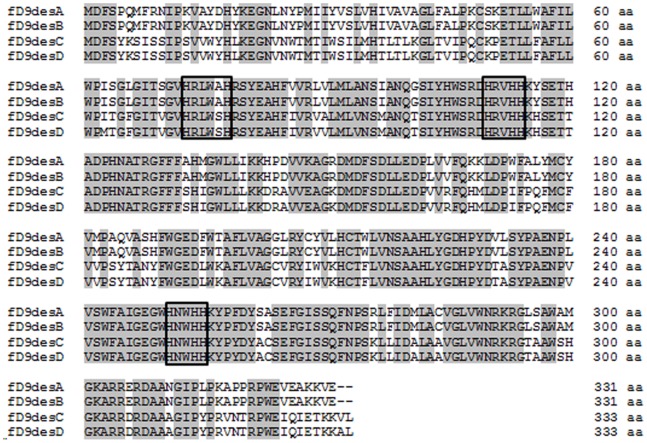
Phylogenetic tree of Δ^9^ desaturases from various organisms. Six putative Δ^9^ desaturase sequences from *Fistulifera* sp. were highlighted. The phylogenetic tree was constructed by the neighbor-joining method using MEGA4 with bootstrap values based on the sequence alignments by the ClustalW program. The query sequences of the representative Δ^9^ desaturase sequences are *P. tricornutum* (GenBank accession number: EEC47008.1), *Acheta domesticus* (AAK2579 7.1), *Ctenopharyngodon idella* (CAB53008.1), *Homo sapiens* (AAD29870.1), *Mus musculus* (AAA40103.1), *Rattus norvegicus* (AAM34745.1), *Synechocystis* sp. PCC 6803 (BAL36622.1), *Synechococcus* sp. PCC 7002 (AAB61353.1), *Nostoc* sp. 36 (desC1) (CAF18423.1), *Gloeobacter violaceus* PCC 7421 (BAC90807.1), *Nostoc* sp. 36 (desC2) (CAF18426.1), *Thermosynechococcus elongates* BP-1 (AAD00699.1), *Asclepias syriaca* (AAC49719.1), *Pelargonium x hortorum* (AAC49421.1), *Ricinus communis* (CAA39859.1), and *Carthamus tinctorius* (AAA33021.1).

**Figure 2:**
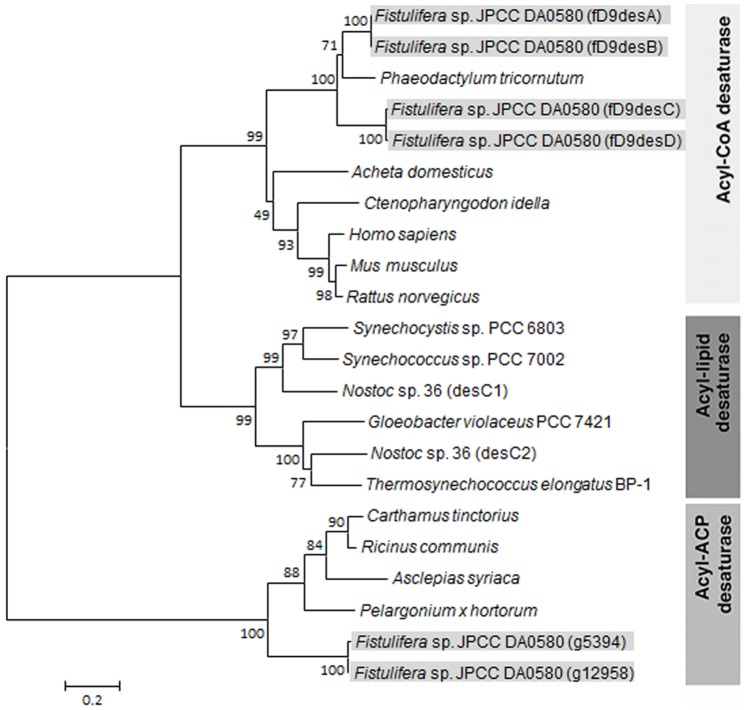
Amino acid sequence alignments of four Δ^9^ desaturases from *Fistulifera* sp. The ClustalW program was used for the alignment. The conserved amino acids are on gray backgrounds. The 3 histidine clusters are framed.

The 6 *Δ^9^ desaturase* candidate genes (Gene ID: g5394, g10778, g10781, g12958, g19483 and g19486) were identified with the BlastX algorithm from all 19,859 genes of this strain as the query sequence from a non-redundant protein sequences database [Manuscript in preparation]. Because the predicted Δ^9^ desaturases from draft genome sequence seemed to be partial ORFs due to the lack of the conserved motifs of histidine box and cytochrome *b_5_* domain, the full-length sequences of putative *acyl-CoA Δ^9^ desaturase* cDNA were sequenced from the products obtained by rapid amplification of cDNA ends (RACE) PCR. The cDNAs containing predicted gene regions were verified to be 996 bp for g10778 and g19483 and 1,002 bp for g10781 and g19486; these were designated as *fD9desA*, *fD9desB fD9desC*, and *fD9desD*, respectively (Fig. 1). The *fD9desA* nucleotide sequences exhibit high identity with the *fD9desB* (96%), and the *fD9desC* had 93% identity with the *fD9desD*. The amino acid sequences of fD9desA and fD9desB were identical, while fD9desC and fD9desD showed the differences of 2 amino acid residues. The 2 proteins encoded by g5394 and g12958 had 49% and 47% identity, respectively, with Δ^9^ desaturase from the plant *Asclepias syriaca* (GenBank accession no. AAC49719.1) [35]. The 4 proteins encoded by g10778, g10781, g19483 and g19486 showed 78%, 69%, 72% and 71% identity, respectively, with the putative Δ^9^ desaturase from a diatom, *P. tricornutum* (EEC47008.1). A phylogenetic tree of Δ^9^ desaturase amino acid sequences from different organisms, prepared using ClustalW, showed that the g5394 and g12958 genes appeared in the cluster of the plastidial acyl-ACP desaturase group, while the remaining genes were categorized in acyl-CoA Δ^9^ desaturase groups localizing in the endoplasmic reticulum (ER) (Fig. 2). As the PUFA synthesis occurs in the ER of eukaryotic cells [19], these 4 genes in the acyl-CoA Δ^9^ desaturase groups were further investigated. In model strains of pennate and centric diatoms, *P. tricornutum* (EEC47008), and *Thalassiosira pseudonana* (EED91785 and EED86245), ER acyl-CoA desaturases and plastidal acyl-ACP desaturases were also identified by a BLAST search using the predicted genes from *Fistulifera* sp. as the query sequence (Table 1).

**Table 1:** Comparison of conserved motifs in Δ9 desaturases from diatoms.

Organism	Gene	AccessionNo.	Conserved histidine sequences			Cytochromeb_5_	Number of predicted TMHs	
	name		First	Second	Third	bidingdomain	TMHMM	HMMTOP
Diatom								
* Fistulifera* sp.	*fD9desA*	AB831011	HRLWAH	HRVHH	HNWHH	Not observed	2	5
	*fD9desB*	AB831012	HRLWSH	HRVHH	HNWHH	Not observed	2	5
	*fD9desC*	AB831013	HRLWAH	HRVHH	HNWHH	Not observed	2	5
	*fD9desD*	AB831014	HRLWSH	HRVHH	HNWHH	Not observed	2	5
* Phaeodactylum tricornutum*	*–*	EEC47008	HRLWSH	HRVHH	HNWHH	Not observed	4	5
* Thalassiosira pseudonana*	*–*	EED91785	HRLWSH	HRVHH	HNWHH	Not observed	3	5
	*–*	EED86245	HRLWSH	HRVHH	HNWHH	Not observed	3	5

A unique histidine motif is generally found in Δ^9^ desaturases [36]. Four *Δ^9^ desaturases* genes in *Fistulifera* sp. possessed 3 histidine motifs (HxxxxH, HxxHH, and HxxHH) involved in the coordination of the di-iron center as the active site of desaturation (Table 1). On the other hand, the cytochrome *b_5_* domain, which is expected to transfer electrons away from the histidine motif, was not observed in the identified Δ^9^ acyl-CoA desaturase. These unique motifs of Δ^9^ desaturases were compared with those in 2 diatoms, *P. tricornutum* and *T. pseudonana* [37]. These enzymes from the 3 diatoms were well conserved, possessing the histidine sequences without the cytochrome *b_5_* domain. The findings concerning the lack of cytochrome *b_5_* in desaturases is in agreement with those of a previous report about alternative desaturation activity in the absence of the cytochrome *b_5_* domain in 2 other desaturases from *T. pseudonana* [38]. Furthermore, the results of truncation and disruption experiments for the cytochrome *b_5_* domain Δ^9^ desaturases have suggested that this domain is not strictly required for fatty acid desaturation [39].

Localization of general Δ^9^ desaturases in the ER membrane are predicted from N-terminal amino acid sequences [19]. Three algorithms for subcellular targeting prediction, TargetP 1.1, SignalP 4.0 and HECTAR, were used in this study. The Δ^9^ desaturase candidates were predicted to have neither N-terminal signal sequences nor internal cleavable signal sequences. All 4 Δ^9^ desaturases were expected to possess 2–5 transmembrane domains, according to a prediction by TMHMM 2.0 and HMMTOP 2.0 (Table 1). This prediction supports the existence of transmembrane helices in Δ^9^ desaturases from *Fistulifera* sp., although further analysis is needed to confirm the number of transmembrane regions.

### Functional Characterization of Putative Δ^9^ Desaturases in the Yeast *Δole1* Mutant

**Figure 3:**
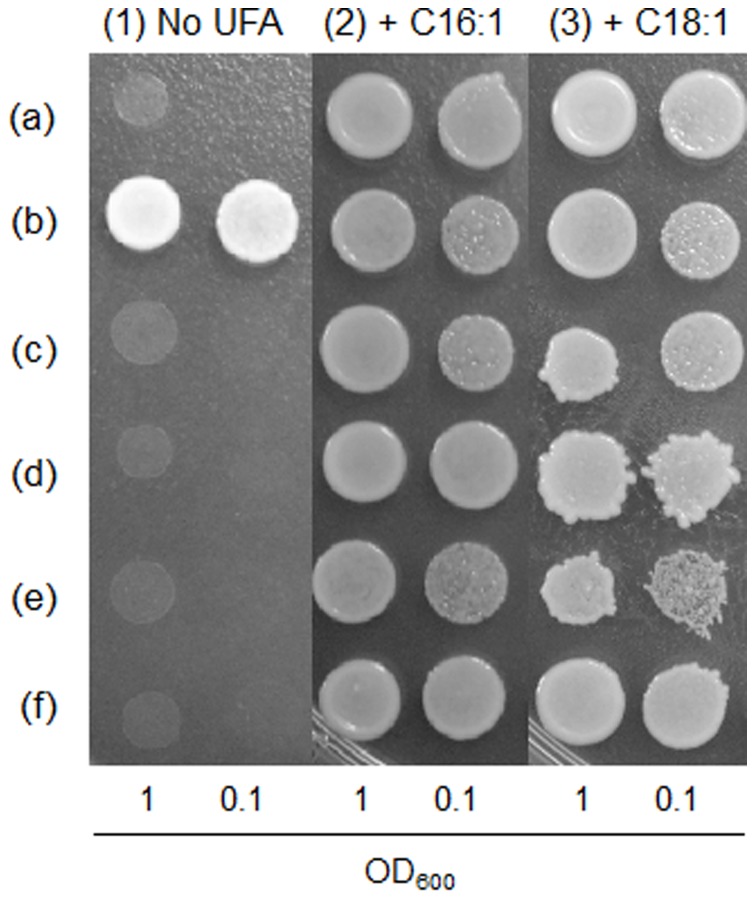
Complementation assay of four Δ^9^ desaturases from *Fistulifera* sp. in the yeast *Δole1* mutant. A dilution series of each yeast sample solution was spotted on SD medium-based agar plates containing galactose and (1) no unsaturated fatty acids (UFAs), (2) 500 µM of the C16∶1 fatty acid, or (3) 500 µM of the C18∶1 fatty acid. (a), the yeast strain INVSc-1 transformed with the control vector pYES2.1/V5-His/lacZ served as a positive control; (b), the yeast *Δole1* mutant transformed with pYES2.1/V5-His/lacZ served as a negative control; (c-f), the yeast *Δole1* mutant transformed with pYES2.1/V5-His/lacZ contained *fD9desA, fD9desB, fD9desC* or *fD9desD* genes.

In order to confirm the function of Δ^9^ desaturases, 4 putative genes were cloned in the protein expression vector, pYES2.1/V5-His-TOPO, and transformed in the yeast *Δole1* mutant. *Δole1* is a *Δ^9^ desaturase* knockout mutant that requires supplementation with C16∶1 or C18∶1 fatty acids to grow. When the putative Δ^9^ desaturases work properly in the synthesis of monounsaturated C16∶1 and/or C18∶1 fatty acids, the transformants can grow on an agar plate without supplementation with exogenous unsaturated fatty acids. The transformed yeast *Δole1* mutant with the control vector (in the absence of any *desaturase* gene), serving as a negative control, did not grow in the absence of fatty acid supplementation (Fig. 3(a), no UFA). The INVSc-1 cells possessing the native *ole1* gene in the genome, serving as a positive control, grew well in the absence of fatty acid supplementation (Fig. 3(b), no UFA) because the cell can generate these essential desaturated fatty acids endogenously. The *fD9desA*, *fD9desB*, *fD9desC*, and *fD9desD* genes failed to complement the yeast *ole1* mutation in the absence of 16∶1/18∶1 fatty acid supplementation (Fig. 3(c)-(f), no UFA). Additionally, the growth of transformants with 16∶1/18∶1 fatty acid supplementation was also confirmed. In a previous study, a Δ^9^ desaturase (Δ9-3) from the fungus *Mortierella alpina* failed to complement the yeast *Δole1* mutant when transformants were grown in the absence of monounsaturated fatty acids supplementation [40]. It is likely that the Δ9-3 desaturase from *M. alpina* and Δ^9^ desaturases from *Fistulifera* sp. did not have sufficient activity for the complementation in yeast, respectively.

Next, to further investigate the *in vivo* function and specificity of these *Δ^9^ desaturase* genes, the fatty acid profiles of the yeast *Δole1* transformant-carrying gene expression vector with the *Δ^9^ desaturase* genes were evaluated by gas chromatography and mass spectrometry (GC/MS) analysis (Table 2). Two Δ^9^ desaturases from *Fistulifera* sp., fD9desC and fD9desD, showed activity for C16∶0 as a substrate (0.6% and 0.3% desaturation to C16∶1, respectively) (100 × product/substrate+product) during growth with C18∶1 supplementation (Table 2) but did not show activity for C18∶0. The remaining 2 Δ^9^ desaturases, fD9desA and fD9desB, showed similar activities to each another with C16∶0 as a substrate (0.8% and 1.2% desaturation to C16∶1, respectively). However, the ideal substrate of fD9desA and fD9desB was C18∶0 converted to C18∶1 (6.9% and 5.5% desaturation to C18∶1). The desaturation efficiencies of fD9desA and fD9desB for C18∶0 were significantly higher than those for C16∶0. The detected value of desaturation by Δ^9^ desaturases from *Fistulifera* sp in yeast was similar to that of house cricket (*Acheta domesticus*) (5% desaturation to 18∶1) [41]. These heterogeneous gene expressions and functional analyses in the model organism may cause the negative effect for the activity, exhibiting these relatively low activities.

**Table 2:** Substrate specificity analysis of Δ^9^ desaturases (fD9desA, fD9desB, fD9desC, and fD9desD) from *Fistulifera* sp. on the basis of expression in the yeast *Δole1* mutant (n = 3).

Transformant	Supplementation of C18∶1		Supplementation of C16∶1	
	C16∶0 (%)^a^	C16∶1 (%)^a^	C18∶0 (%)^a^	C18∶1 (%)^a^
INVSc-1+ control vector	27.2±3.1	57.2±7.4	26.4±1.9	7.9±0.6
Δ*ole1*+ control vector	65.6±0.4	0	31.7±1.8	0
*Δole1*+ fD9desA	72.1±2.4	0.6±0.2	24.7±0.7	1.8±0.2
*Δole1*+ fD9desC	70.4±3.1	0.9±0.3	27.5±2.2	1.6±0.3
*Δole1*+ fD9desB	72.4±3.2	0.5±0.5	27.7±0.9	0
*Δole1*+ fD9desD	69.6±2.1	0.2±0.1	28.5±1.7	0

The yeast strain INVSc-1 transformed with the control vector pYES2.1/V5-His/*lacZ* (Control vector) served as the positive control. The yeast *Δole1* mutant transformed with pYES2.1/V5-His/*lacZ* served as the negative control.

a The relative amount of each fatty acid was expressed as a percentage of total fatty acids (± SD) after subtracting the amount of the supplemented fatty acid from the total.

## Discussion

Six *Δ^9^ desaturase* genes were identified in *Fistulifera* sp. by bioinformatic analysis. These genes were categorized as 4 ER acyl-CoA desaturases (*fD9desA*, *fD9desB*, *fD9desC*, and *fD9desD*) and 2 plastidial acyl-ACP desaturases (g5394 and g12958). By way of comparison, *P. tricornutum* has only 1 ER acyl-CoA desaturase and 1 plastidial acyl-ACP desaturase, and *T. pseudonana* has 2 ER acyl-CoA desaturases and 1 plastidial acyl-ACP desaturase (Table 1). These results indicate that *Fistulifera* sp. has more *Δ^9^ desaturase* genes than other diatoms do. The higher number of *Δ^9^ desaturase* genes in *Fistulifera* sp. suggests that the oleaginous strain may have well-developed genome organization for fatty acid metabolism to enable considerable accumulation of TAG endogenously. The identified ER acyl-CoA desaturases show different substrate specificities (Table 2). Two Δ^9^ desaturases (fD9desA and fD9desB) converted both C16∶0 and C18∶0 to C16∶1 and C18∶1, respectively, and the other 2 Δ^9^ desaturases (fD9desC and fD9desD) converted C16∶0 to C16∶1. The sequences of the Δ^9^ desaturases showed the presence of highly conserved histidine cluster motifs [42] (Fig. 2), and a difference of 1 amino acid residue in the first histidine motif box was observed among these 4 Δ^9^ desaturases. The former group of Δ^9^ desaturases (fD9desA and fD9desB) has the HRLWSH sequence, and the latter group of Δ^9^ desaturases (fD9desC and fD9desD) has the HRLWAH sequence, with the serine substituted with alanine. In other words, Δ^9^ desaturase pairs from *Fistulifera* sp. have the same histidine motifs and desaturation specificities (Table 2). In the oil-producing fungus *M. alpina,* a different amino acid sequence in the first histidine box was found in 3 Δ^9^ desaturases. Different Δ^9^ desaturases with different sequences in the first histidine box showed varying specificities for fatty acid desaturation [40]. Therefore, the difference in amino acid sequences is considered to reflect the substrate specificity of Δ^9^ desaturases from *Fistulifera* sp. *P. tricornutum* has only 1 ER acyl-CoA desaturase with the amino acid sequence of HRLWSH in the first histidine motif, while *T. pseudonana* has 2 ER acyl-CoA desaturases with the same amino acid sequence (HRLWSH; Table 1). On the basis of these results, we hypothesized that all the Δ^9^ desaturases in both diatoms possess the same substrate specificity, although a detailed functional analysis should be performed to confirm this hypothesis. If it is confirmed, this would indicate that the varying specificities of Δ^9^ desaturases from *Fistulifera* sp. may be related to the specific fatty acid metabolism in this strain. In addition, the Δ^9^ desaturation activity was assayed for the substrates of 16∶0 and 18∶0. In the case of mouse Δ^9^ desaturase, for wide range of saturated fatty acids from 12∶0 to 19∶0, the desaturation activity was detected [43]. To fully confirm the functional specificity of Δ^9^ desaturase from *Fistulifera* sp. for various substrates, further analysis should be provided in the future.

In conclusion, 4 ER *Δ^9^ acyl-CoA desaturase* and 2 plastidial *Δ^9^ acyl-ACP desaturase* genes were identified from the oleaginous diatom *Fistulifera* sp., and gene homologs were also observed in other diatoms. Among the *Δ^9^ desaturase* genes, four *Δ^9^ acyl-CoA desaturases* showed desaturation activity of the saturated fatty acids of C16∶0 and/or C18∶0 in complementation assays using the yeast *Δole1* mutant. This study is the first functional confirmation of Δ^9^ desaturase from diatom and from oleaginous microalgae. While the *in vivo* function of the *Δ^9^ desaturase* genes in the *Fistulifera* sp. should be separately addressed, this study provides information that will help in the regulation of the fatty acid profiles in the oleaginous microalgae.

## Supporting Information

Table S1 **List of primers used for this study.**
(DOCX)
