# Supplementary material for: Identification and Functional Analysis of Delta-9 Desaturase, a Key Enzyme in PUFA Synthesis, Isolated from the Oleaginous Diatom Fistulifera
Source: PLoS One. 2013 Sep 5;8(9):e73507. doi: 10.1371/journal.pone.0073507 (PMC3764056; doi:10.1371/journal.pone.0073507)
Supplement: Table S1 — List of primers used for this study. (DOCX) [file pone.0073507.s001.docx]

| Gene | Primer name | Sequence 5'-3' |
| --- | --- | --- |
| Primers used for 5' and 3' RACE amplification | | |
| *fD9desA* | g19483-5'-RACE | TAGAGAGCAAACCAGGGATCCAGCTTCT |
|  | g19483-5'-Nested | AGGGATCTTCGAGCAAGTCAGAGA |
|  | g19483-3'-RACE | CTACGTGTTGCACTGCACTTGGCTCG |
| *fD9desB* | g10778-5'-RACE | GTAGAGTGCAAACCAGGGATCAAGCTTC |
|  | g10778-5'-Nested | AAGGGGATCTTCGAGCAAGTCAGAG |
|  | g10778-3'-RACE | CCGCTCAAGTCGCGTCACACTTCTG |
| *fD9desC* | g19486-5'-RACE | GCCGAGTTCACCAAGAATGTGCAATGCT |
|  | g19486-5'-Nested | TGACCCAGATGTAGCGCACACAA |
|  | g19486-3'-RACE | ATCTCTGGAAAGCGTTCTTGGTTGCTGG |
| *fD9desD* | g10781-5'-RACE | GTGAGAAAAGAAGAAGCCACGTGTTGCG |
|  | g10781-5'-Nested | TGTGAGGATCAGCAGTAGTTTCCG |
|  | g10781-3'-RACE | CATTCTTGGTAAACTCGGCGGCGCAC |
|  | g10781-3'-Nested | GTATGGTGATCATCCGTACGACAC |
| Primers used for full-length cloning and yeast transformations | | |
| *fD9desA* | g19483Fw | CCATTTATTCTATAATTATCGACAATATG |
|  | g19483Rv | CTCGACTTTCTTGGCTTCGA |
| *fD9desB* | g10778Fw | GATTCAACAACTACCGACAATATG |
|  | g10778Rv | CTCGACTTTCTTGGCTTCGA |
| *fD9desC* | g19486Fw | TTGTCTATACTTCATCATCATCATG |
|  | g19486Rv | CAGGACCTTCTTGGTTTCAATC |
| *fD9desD* | g10781Fw | CCTTGTCTATACTTCATCATCATG |
|  | g10781Rv | CAGGGCCTTCTTGGTTTCGA |

**Table S1 List of primers used for this study**
